# Presepsis biomarker: high-density lipoprotein

**DOI:** 10.1186/cc14080

**Published:** 2014-12-03

**Authors:** S Al-Zaidawi

**Affiliations:** 1 Surgery Department, Burn Unit, Al-Sadr Teaching Hospital, Maysan, Iraq

## Introduction

Delay in diagnosis and initiation of antibiotic treatment has been shown to increase mortality. Biomarkers can play an important role in diagnosis and prognosis of sepsis. We aimed to evaluate the correlation between septicemia and high-density lipoprotein (HDL) level in burned patients.

## Methods

A prospective study conducted at Al-Sadr teaching hospital, Maysan, Iraq, during the period from April to September 2013. Blood samples were collected from patients every other day to measure the level of HDL and triglycerides. Other blood samples were collected in blood culture tubes for culturing to verify septicemia depending on the clinical evidence.

## Results

Seventy-five patients (Table 1) were admitted consecutively into the burn unit, 35 of them (46%) developed septicemia and 11 of the 35 patients died. All dead patients had HDL value <5 mg/dl 1 or 2 days before dying since our blood samples were collected every 2 days (Tables 2 and 3). Other laboratory tests such as WBCs, platelet account, albumin level, and so forth were made to confirm sepsis (Table 4). A comparison between the level of lipid profile before and after sepsis showed a significant drop in HDL level during the onset of sepsis (Table 5). We also found that patients with HDL value <15 mg/dl were at high risk of developing sepsis.

**Table 1:** Characteristics of patients

Patient characteristic	Average	Range
Age (years)	17	1 to 85
TBSA%	33.5%	15 to 95%
Sex of patients	Female 61%	Male 39%
Burn type	Scalds 48%	Flame 52%

Most patients were female (61%) with average age 17 years and a wide range of burned surface area (15 to 95%)

**Table 2:** Lipid profile for all 75 patients at the onset of thermal injury during the first day of admission: all patients were with a normal range of HDL, triglycerides and cholesterol

Lipid profile	Range (mg/dl)	Average (mg/dl)	Mode (mg/dl)	Normal range (mg/dl)
HDL	30 to 56	39	38	39 to 59
Triglycerides	37 to 148	70	58	0 to 149
Cholesterol	46 to 155	78	86	0 to 199

**Table 3:** Lipid profile at the onset of sepsis showed that HDL level dropped to less than 15 mg/dl with range (4 to 13 mg/dl): elevation in triglyceride level out of normal range with no significant change in cholesterol level

Lipid profile	Range (mg/dl)	Average (mg/dl)	Mode (mg/dl)	Normal range (mg/dl)
HDL	4 to 13	7.6	4	39 to 59
Triglycerides	133 to 435	214.5	180	0 to 149
Cholesterol	56 to 139	82.8	76	0 to 199

**Table 4:** Levels of urea, creatinine, albumin, WBC, platelets during onset of sepsis: most patients developed hypoalbuminemia and thrombocytopenia

	%TBSA	WBC (×1,000/μl)	Platelet count (×1,000/μl)	Blood urea (mg/dl)	Serum creatinine	Serum albumin (g/dl)	Time to get sepsis (days)
Average	55	11.86	154.9	14	0.51	2.0	7
Mode	45	9.04	121	12	0.37	1.8	10
Minimum	27	2.27	32	9	0.32	1.4	2
Maximum	95	15.03	535	21	0.73	3.1	20

**Table 5:** Comparison between level of lipid profile before and after sepsis showed the significant dropping in HDL level during onset of sepsis

	HDL	Triglycerides	Cholesterol	*P *value
Burn onset	39	70	78	< 0.01

Sepsis onset	7.6	214.5	76	< 0.01

## Conclusion

There was a strong correlation between HDL level and septicemia in burn patients. The HDL value is a good biomarker for sepsis; it decreases below normal level and continues to diminish and reach an immeasurable level at the advanced stage of septicemia.

